# Supplementary material for: Unraveling the mouse model of Staphylococcus aureus bacteremia and sepsis: a systematic approach to better characterize host/pathogen interactions
Source: Microbiol Spectr. 2026 Jan 20;14(3):e02642-25. doi: 10.1128/spectrum.02642-25 (PMC12955411; doi:10.1128/spectrum.02642-25)
Supplement: Supplemental figures and tables — Figure S1 and Tables S1 to S6. [file spectrum.02642-25-s0001.docx]

**Fig. S1.** **Schematic diagram summarizing the mouse model.** Eight-week-old female specific-pathogen free (SPF) CD-1 or C57BL/6N mice were infected with 0.5-1.0 x 10^7 CFU of each *S. aureus* strain by injection into the tail vein (time 0) then monitored for 9 (SPF CD-1 mice) or 2 (C57BL/6N mice) days. Blood and/or organs (kidneys and liver) collections were performed at the indicated time points. IV: intravenous.

**Figure Supplementary 1**


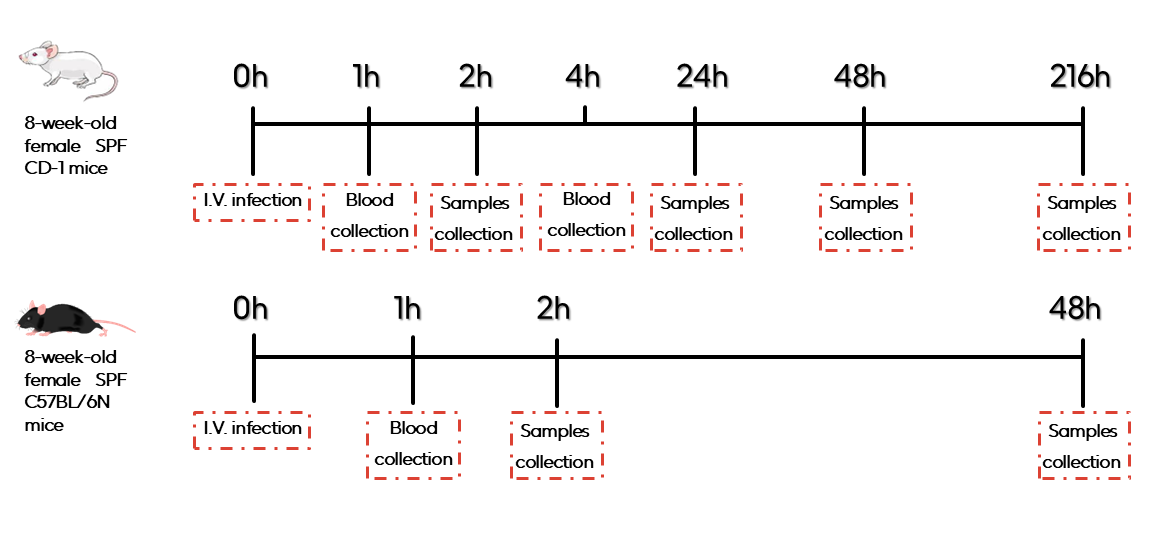


**Table ST1**. **CFU counts in the blood of *S. aureus* infected CD-1 mice at different time points.** Data from 8 to 22 animals and 4 independent experiments have been collected at each time point. Mean and 95% coefficient interval (CI) range of log10 CFU counts for each condition were reported.

| **STRAIN** | **TIME POINT (hours)** | **MEAN LOG10 CFU** | **Lower 95% CI** | **Upper 95% CI** | **N° colonized mice/ N° infected mice** |
| --- | --- | --- | --- | --- | --- |
| LAC (USA300 – CC8) | 1 | 2.76 | 2.63 | 2.89 | 14/14 |
| LAC (USA300 – CC8) | 2 | 2.71 | 2.50 | 2.91 | 22/22 |
| LAC (USA300 – CC8) | 4 | 2.75 | 2.55 | 2.96 | 10/10 |
| LAC (USA300 – CC8) | 24 | 2.13 | 1.66 | 2.60 | 18/22 |
| LAC (USA300 – CC8) | 48 | 1.42 | 0.95 | 1.90 | 10/13 |
| LAC (USA300 – CC8) | 216 | 0.86 | 0.49 | 1.23 | 5/8 |
| MW2 (USA400 – CC1) | 1 | 2.71 | 2.59 | 2.83 | 13/13 |
| MW2 (USA400 – CC1) | 2 | 2.93 | 2.85 | 3.00 | 22/22 |
| MW2 (USA400 – CC1) | 4 | 3.16 | 3.04 | 3.28 | 10/10 |
| MW2 (USA400 – CC1) | 24 | 2.28 | 1.88 | 2.68 | 21/22 |
| MW2 (USA400 – CC1) | 48 | 2.01 | 1.57 | 2.46 | 13/14 |
| MW2 (USA400 – CC1) | 216 | 1.14 | 0.69 | 1.58 | 6/8 |
| μ50 (USA100 – CC5) | 1 | 2.65 | 2.56 | 2.75 | 14/14 |
| μ50 (USA100 – CC5) | 2 | 2.30 | 2.06 | 2.54 | 22/22 |
| μ50 (USA100 – CC5) | 4 | 2.66 | 2.36 | 2.97 | 10/10 |
| μ50 (USA100 – CC5) | 24 | 1.66 | 1.13 | 2.19 | 18/22 |
| μ50 (USA100 – CC5) | 48 | 0.91 | 0.44 | 1.39 | 14/22 |
| μ50 (USA100 – CC5) | 216 | 0.44 | 0.34 | 0.54 | 1/8 |
| TW20 (ST239 – CC8) | 1 | 3.29 | 3.19 | 3.39 | 14/14 |
| TW20 (ST239 – CC8) | 2 | 2.97 | 2.63 | 3.30 | 22/22 |
| TW20 (ST239 – CC8) | 4 | 3.39 | 3.22 | 3.56 | 10/10 |
| TW20 (ST239 – CC8) | 24 | 1.68 | 1.30 | 2.05 | 19/22 |
| TW20 (ST239 – CC8) | 48 | 1.84 | 1.22 | 2.46 | 12/14 |
| TW20 (ST239 – CC8) | 216 | 0.97 | -0.01 | 1.95 | 3/8 |

**Table ST2**. **CFU counts in the kidneys of *S. aureus* infected CD-1 mice at different time points.** Data from 8 to 16 animals and 3 independent experiments have been collected at each time point. Mean and 95% coefficient interval (CI) range of log10 CFU counts for each condition were reported.

| **STRAIN** | **TIME POINT (hours)** | **MEAN LOG10 CFU** | **Lower 95% CI** | **Upper 95% CI** |
| --- | --- | --- | --- | --- |
| LAC (USA300 – CC8) | 2 | 3.08 | 2.60 | 3.56 |
| LAC (USA300 – CC8) | 24 | 4.13 | 3.55 | 4.71 |
| LAC (USA300 – CC8) | 48 | 5.43 | 3.91 | 6.94 |
| LAC (USA300 – CC8) | 216 | 6.36 | 4.15 | 8.57 |
| MW2 (USA400 – CC1) | 2 | 3.56 | 2.81 | 4.32 |
| MW2 (USA400 – CC1) | 24 | 5.98 | 5.63 | 6.33 |
| MW2 (USA400 – CC1) | 48 | 7.56 | 6.37 | 8.76 |
| MW2 (USA400 – CC1) | 216 | 8.61 | 7.87 | 9.34 |
| μ50 (USA100 – CC5) | 2 | 2.53 | 1.79 | 3.27 |
| μ50 (USA100 – CC5) | 24 | 2.31 | 1.91 | 2.71 |
| μ50 (USA100 – CC5) | 48 | 2.13 | 0.29 | 3.97 |
| μ50 (USA100 – CC5) | 216 | 2.34 | 2.02 | 2.65 |
| TW20 (ST239 – CC8) | 2 | 3.86 | 3.43 | 4.29 |
| TW20 (ST239 – CC8) | 24 | 3.72 | 3.15 | 4.28 |
| TW20 (ST239 – CC8) | 48 | 4.81 | 3.68 | 5.94 |
| TW20 (ST239 – CC8) | 216 | 4.57 | 2.81 | 6.32 |

**Table ST3**. **CFU counts in the liver of *S. aureus* infected CD-1 mice at different time points.** Data from 8 animals and 2 independent experiments have been collected at each time point. Mean and 95% coefficient interval (CI) range of log10 CFU counts for each condition were reported.

| **STRAIN** | **TIME POINT (hours)** | **MEAN LOG10 CFU** | **Lower 95% CI** | **Upper 95% CI** |
| --- | --- | --- | --- | --- |
| LAC (USA300 – CC8) | 2 | 5.80 | 5.47 | 6.15 |
| LAC (USA300 – CC8) | 24 | 3.86 | 3.48 | 4.24 |
| LAC (USA300 – CC8) | 48 | 3.67 | 3.21 | 4.13 |
| LAC (USA300 – CC8) | 216 | 3.21 | 2.71 | 3.72 |
| MW2 (USA400 – CC1) | 2 | 5.83 | 5.26 | 6.40 |
| MW2 (USA400 – CC1) | 24 | 4.16 | 3.78 | 4.55 |
| MW2 (USA400 – CC1) | 48 | 4.74 | 3.73 | 5.75 |
| MW2 (USA400 – CC1) | 216 | 4.49 | 3.13 | 5.86 |
| μ50 (USA100 – CC5) | 2 | 5.95 | 5.65 | 6.25 |
| μ50 (USA100 – CC5) | 24 | 4.17 | 3.75 | 4.59 |
| μ50 (USA100 – CC5) | 48 | 2.53 | 1.98 | 3.09 |
| μ50 (USA100 – CC5) | 216 | 2.96 | 1.93 | 4.00 |
| TW20 (ST239 – CC8) | 2 | 6.10 | 5.91 | 6.27 |
| TW20 (ST239 – CC8) | 24 | 3.90 | 3.61 | 4.18 |
| TW20 (ST239 – CC8) | 48 | 4.16 | 3.83 | 4.50 |
| TW20 (ST239 – CC8) | 216 | 4.23 | 2.44 | 6.03 |

**Table ST4: CFU counts in the blood of *S. aureus* infected C57BL/6N mice at different time points.** Data from 3 independent experiments and 12 mice/groups were reported as mean ± 95% coefficient interval (CI) range of log10 CFU counts for each time point.

| **STRAIN** | **TIME POINT (hours)** | **MEAN LOG10 CFU counts**  **± 95 % C.I.** | **N° colonized mice/ N° infected mice** |
| --- | --- | --- | --- |
| LAC (USA300 – CC8) | 1 | 2.77 ± 0.22 | 11/11 |
| LAC (USA300 – CC8) | 2 | 2.84 ± 0.14 | 12/12 |
| LAC (USA300 – CC8) | 48 | 1.55 ± 0.52 | 9/11 |
| MW2 (USA400 – CC1) | 1 | 2.85 ± 0.14 | 12/12 |
| MW2 (USA400 – CC1) | 2 | 2.98 ± 0.09 | 12/12 |
| MW2 (USA400 – CC1) | 48 | 1.93 ± 0.26 | 12/12 |
| μ50 (USA100 – CC5) | 1 | 2.53 ± 0.15 | 12/12 |
| μ50 (USA100 – CC5) | 2 | 2.31 ± 0.17 | 12/12 |
| μ50 (USA100 – CC5) | 48 | 1.05 ± 0.47 | 4/12 |
| TW20 (ST239 – CC8) | 1 | 3.08 ± 0.10 | 12/12 |
| TW20 (ST239 – CC8) | 2 | 2.88 ± 0.12 | 12/12 |
| TW20 (ST239 – CC8) | 48 | 1.73 ± 0.77 | 8/12 |

**Table ST5: Bacteria load in the kidneys of C57BL/6N mice** **infected with different epidemiologically relevant *S. aureus* strains.** Data from 3 independent experiments and 12 mice/groups were reported as mean ± 95% coefficient interval (CI) range of log_10_ CFU counts for each time point.

| **STRAIN** | **MEAN LOG_10_ CFU counts ± 95 % C.I.**  **(2h post-infection)** | **MEAN LOG_10_ CFU counts ± 95 % C.I.**  **(48h post-infection)** |
| --- | --- | --- |
| **LAC (USA300 -CC8)** | 3.26 ± 0.17 | 6.14 ± 1.43 |
| **MW2 (USA400 – CC1)** | 3.84 ± 0.18 | 7.60 ± 0.33 |
| **μ50 (USA100 – CC5)** | 3.24 ± 0.25 | 3.53 ± 1.13 |
| **TW20 (ST239 – CC8)** | 3.68 ± 0.10 | 4.82 ± 1.25 |

**Table ST6: Bacteria load in the liver of C57BL/6N mice infected with different epidemiologically relevant *S. aureus* strains.** Data from 3 independent experiments and 12 mice/groups were reported as mean ± 95% coefficient interval (CI) range of log10 CFU counts for each time point.

| **STRAINS** | **MEAN LOG10 CFU counts ± 95 % C.I.**  **(2h post-infection)** | **MEAN LOG10 CFU counts ± 95 % C.I.**  **(48h post-infection)** |
| --- | --- | --- |
| **LAC (USA300 -CC8)** | 6.24 ± 0.28 | 3.64 ± 0.79 |
| **MW2 (USA400 – CC1)** | 6.08 ± 0.21 | 6.79 ± 0.82 |
| **μ50 (USA100 – CC5)** | 6.14 ± 0.24 | 3.04 ± 0.63 |
| **TW20 (ST239 – CC8)** | 6.36 ± 0.28 | 4.26 ± 1.23 |
